# Physiological impact and disease reversion for the severe form of centronuclear myopathy linked to dynamin

**DOI:** 10.1172/jci.insight.137899

**Published:** 2020-09-17

**Authors:** Xènia Massana Muñoz, Christine Kretz, Roberto Silva-Rojas, Julien Ochala, Alexia Menuet, Norma B. Romero, Belinda S. Cowling, Jocelyn Laporte

**Affiliations:** 1 Institut de Génétique et de Biologie Moléculaire et Cellulaire, Illkirch, France.; 2 Centre National de la Recherche Scientifique, UMR7104, Illkirch, France.; 3 Institut National de la Santé et de la Recherche Médicale, U1258, Illkirch, France.; 4 Université de Strasbourg, Illkirch, France.; 5 Centre of Human and Applied Physiological Sciences, School of Basic and Medical Biosciences, Faculty of Life Sciences and Medicine, King’s College London, London, United Kingdom.; 6 Neuromuscular Morphology Unit, Myology Institute, GHU Pitié-Salpêtrière, Paris, France.; 7 Sorbonne Université, AP-HP, INSERM, Centre de référence des maladies neuromusculaires Nord/Est/Ile de France, Paris, France.; 8 Dynacure, Illkirch, France.

**Keywords:** Muscle Biology, Therapeutics, Genetic diseases, Muscle, Neuromuscular disease

## Abstract

Classical dynamins are large GTPases regulating membrane and cytoskeleton dynamics, and they are linked to different pathological conditions ranging from neuromuscular diseases to encephalopathy and cancer. Dominant dynamin 2 (DNM2) mutations lead to either mild adult onset or severe autosomal dominant centronuclear myopathy (ADCNM). Our objectives were to better understand the pathomechanism of severe ADCNM and test a potential therapy. Here, we created the *Dnm2*^SL/+^ mouse line harboring the common S619L mutation found in patients with severe ADCNM and impairing the conformational switch regulating dynamin self-assembly and membrane remodeling. The *Dnm2*^SL/+^ mouse faithfully reproduces severe ADCNM hallmarks with early impaired muscle function and force, together with myofiber hypotrophy. It revealed swollen mitochondria lacking cristae as the main ultrastructural defect and potential cause of the disease. Patient analysis confirmed this structural hallmark. In addition, DNM2 reduction with antisense oligonucleotides after disease onset efficiently reverted locomotor and force defects after only 3 weeks of treatment. Most histological defects including mitochondria alteration were partially or fully rescued. Overall, this study highlights an efficient approach to revert the severe form of dynamin-related centronuclear myopathy. These data also reveal that the dynamin conformational switch is key for muscle function and should be targeted for future therapeutic developments.

## Abstract 2

The dynamin 2 S619L mouse model displays defects in skeletal muscle that are rescued by reducing dynamin 2 protein levels with antisense oligonucleotide treatment.

## Graphical abstract

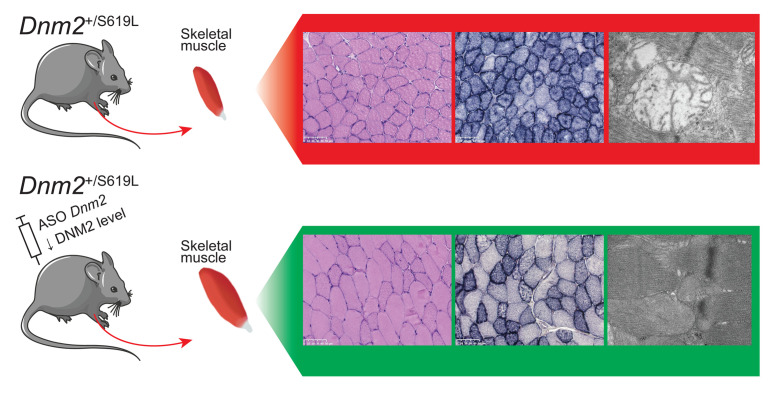

## Introduction

Maintenance and remodeling of the intracellular organization is controlled by cytoskeletons and membrane dynamics. Dynamins are mechanochemical GTPases that catalyze membrane remodeling and control actin polymerization (1–3). Within the classical dynamins, dynamin 2 (DNM2) is the only member ubiquitously expressed. Dominant *DNM2* mutations cause different genetic diseases affecting different tissues: centronuclear myopathy (MIM #160150) (4), Charcot-Marie-Tooth peripheral neuropathy (CMT; MIM #606482) (5) and spastic paraplegia (6). A recessive *DNM2* mutation was also reported in a lethal congenital contracture syndrome (MIM #615368) (7).

Autosomal dominant centronuclear myopathy (ADCNM) due to *DNM2* mutations is characterized by progressive muscle weakness, mainly proximal, and facial weakness with ptosis (4, 8). Histological hallmarks are type I fiber predominance and hypotrophy, along with a general intracellular disorganization with nuclei centrally located and radial organization of the sarcoplasmic reticulum (9). The age of onset and severity are highly heterogeneous, ranging from the severe neonatal onset form to a mild form with adult onset. The most common mutations in the severe and the adult forms are the S619L and R465W missense mutations, respectively (10). Patients with the S619L mutation have a generalized hypotonia at birth associated with ventilator distress requiring mechanical ventilation, which is not observed for the R465W mutation (11). The pathomechanism is still unclear, and the role of DNM2 in muscle is not well understood. Several in cellulo and in vivo models have been created to shed light on DNM2 physiopathological functions and suggest the disease arises from T-tubule fragmentation and a defect in intracellular organization (12–16). The only genetically stable physiological model is the *Dnm2*^RW/+^ mouse (R465W mutation) that displays a mild phenotype with force reduction from 3 weeks (3w), as well as muscle atrophy and abnormal oxidative staining from 2 months of age (17). These defects are rather weak and slightly progressive, with no impact on life span and body weight. To date, no stable animal model for the severe form of ADCNM has been reported. Several *DNM2* ADCNM mutations were exogenously overexpressed in *drosophila*, zebrafish, and mice. *Drosophila* overexpressing S619L DNM2 did not develop to adult stage due to an eclosion defect, and larvae displayed T-tubule disorganization (13). Zebrafish transiently overexpressing S619L DNM2 had decreased movement and structural defects in T-tubules and neuromuscular junctions (NMJs) (12, 15, 18). In mice, several *DNM2* mutations linked to ADCNM were transiently overexpressed and correlated with CNM-like histological defects (14, 16). A faithful model is, thus, still needed to study the pathomechanisms and test potential therapies, especially for the severe form.

No therapies are yet available for any ADCNM forms. Palliative treatment with acetylcholine inhibitors showed improvement of motor behavior in zebrafish larvae and muscle strength and fatigability in patients (18, 19). First proof-of-concept results with prospective technologies such as RNA trans-splicing and RNA inhibition were recently reported. RNA trans-splicing was only tested in WT mice (20), while RNA inhibition with allele-specific shRNA silencing (21) or antisense oligonucleotides (ASO) targeting the total pool of *Dnm2* both prevented the progression of the phenotypes of the *Dnm2*^RW/+^ mouse (22, 23)

*DNM2* mutations in ADCNM are proposed to be gain-of-function mutations. The functions of DNM2 depend on its ability to hydrolyze GTP, oligomerize, and bind lipids (24). In vitro, DNM2 ADCNM mutants increased protein oligomerization and its GTPase activity independently of lipid binding (25, 26). Moreover, overexpression of WT DNM2 in mice induced a CNM-like phenotype, milder than overexpressing the R465W mutant (14, 27).

**Figure 1:**
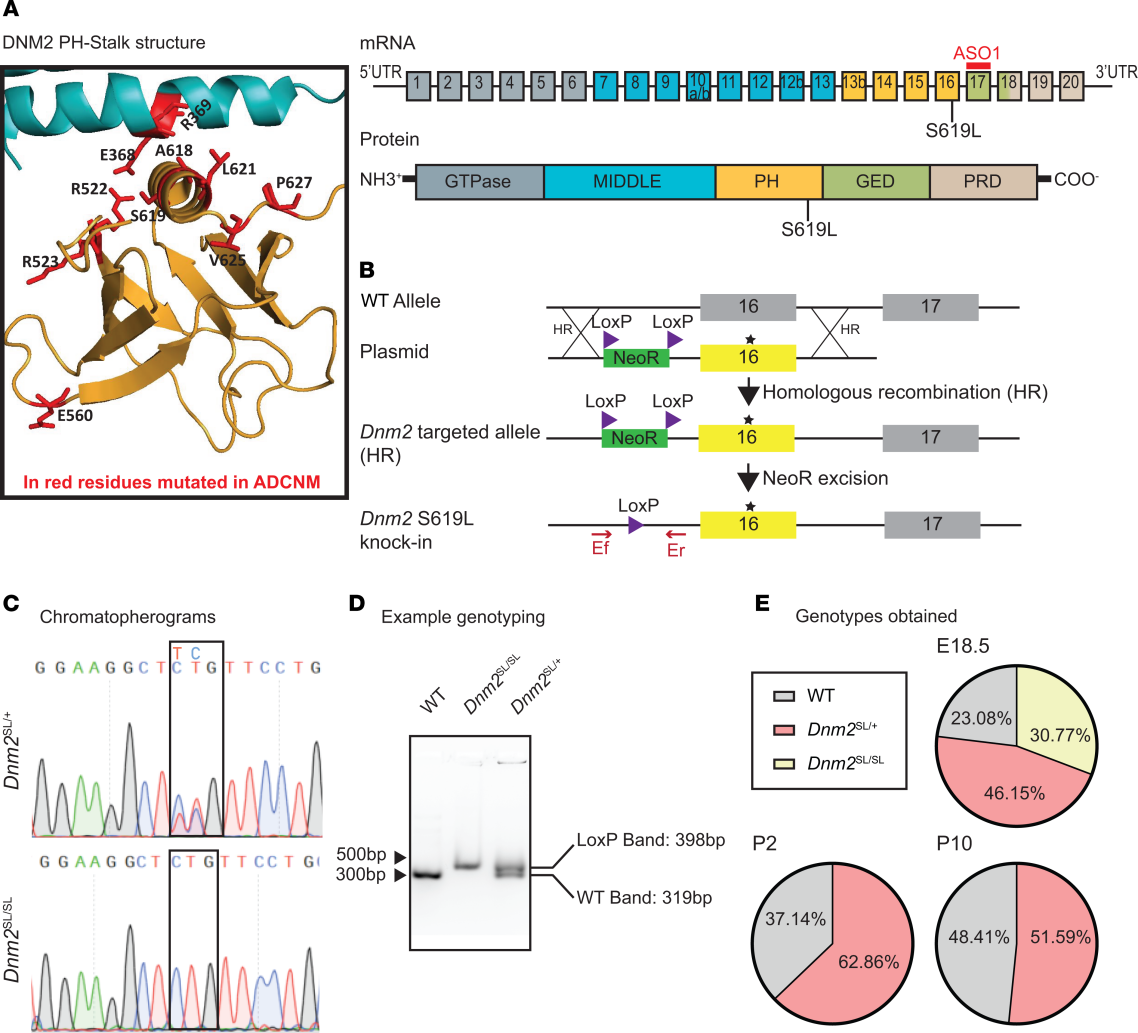
Creation and validation of the *Dnm2*^SL/+^ mouse with the S619L *Dnm2* mutation. (**A**) Left: Structure of the PH-stalk interface in a closed conformation modeled from the structure of dynamin 3 tetramer (PDB ID 5A3F). Residues mutated in ADCNM are in red. The S619 residue is located on the PH domain at the PH-stalk interface. Right: Exonic and protein domain structure of DNM2. ASO-1 is an antisense oligonucleotide targeting murine *Dnm2*. Exons 10a, 10b, 12b, and 13b are alternatively spliced exons. PH, pleckstrin homology; GED, GTPase effector domain; PRD, proline rich domain. The middle and GED domains form the stalk. (**B**) Genomic region, plasmid, and strategy for homologous recombination. (**C**) Chromatopherograms of the *Dnm2* mutation identified in heterozygous and homozygous mice. (**D**) Example of DNA genotyping. (**E**) Proportion of genotypes obtained at 18.5 dpc (*n* = 15), day 2 (P2; *n* = 35), and day 10 (P10; *n* = 130).

Classical dynamins are composed of a GTPase domain, a middle domain, and a GTPase effector domain (GED), which form the stalk and are involved in protein oligomerization; a pleckstrin homology (PH) domain, which binds membranes; and a C-terminal proline rich domain (PRD), which binds SH3-containing proteins (Figure 1A) (28, 29). Most ADCNM DNM2 mutations linked to the severe form, including the S619L, are concentrated in the PH-stalk interface, while the mild R465W mutation does not affect this interface. This interface regulates a conformational switch affecting dynamin self-assembly, membrane binding, and fission (24, 30). The PH-stalk interface in the closed conformation is autoinhibitory, and the release of the PH domain upon membrane binding leads to an open conformation. Several ADCNM mutations weaken this interface, and the severe S619L mutant disrupts this interface, which leads to decreased autoinhibition and aberrant oligomerization in vitro (Figure 1A) (24). However, a modest impact of S619L on endocytosis was noted in culture cells (31), and the physiological impact of this conformational switch has never been investigated.

Here, we aimed to create and validate a genetically stable mammalian model for the severe ADCNM form to study the disease mechanisms and have the opportunity to test potential therapies in a physiological relevant model. We established the *Dnm2*^SL/+^ mouse harboring the S619L *Dnm2* mutation, and we showed that it faithfully reproduces a severe CNM phenotype that was reversed by DNM2 reduction upon ASO treatment. Importantly, this model allows us to study the physiological impact of the conformational switch regulating the functions of DNM2, a protein critical to many cell functions.

## Results

### Creation and validation of the Dnm2^SL/+^ mouse harboring the S619L Dnm2 mutation.

**Figure 2:**
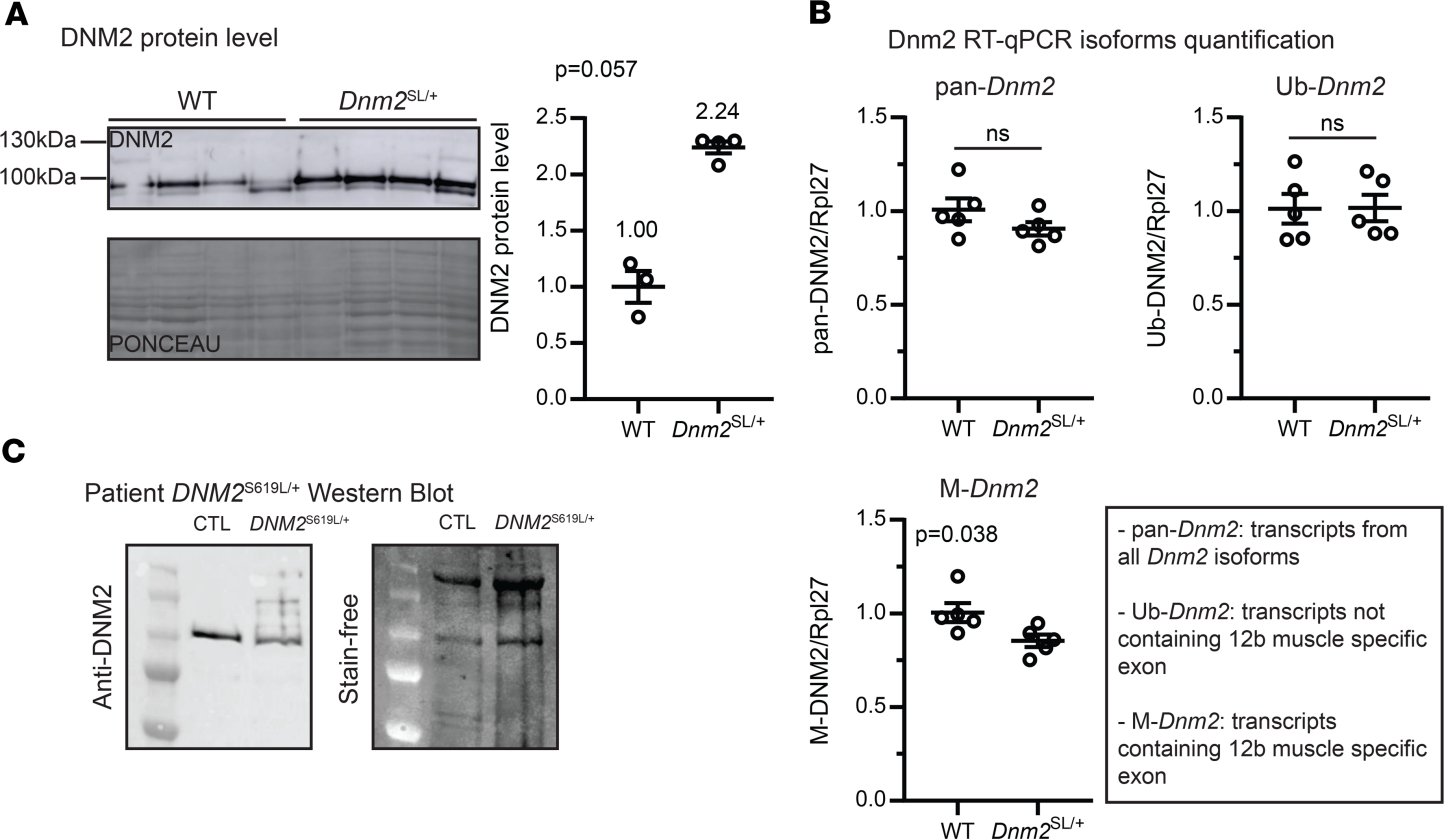
*Dnm2*^SL/+^ animals display an increased DNM2 level. (**A**) DNM2 protein level in TA muscles of 8-week-old WT and *Dnm2*^SL/+^ (3 ≤ *n* ≤ 5; Mann-Whitney *U* test). (**B**) *Dnm2* isoforms quantification. Pan-*Dnm2* corresponds to all isoforms, Ub-*Dnm2* to ubiquitous isoforms not including the muscle-specific exon 12b, M-*Dnm2* to isoforms including the muscle-specific exon 12b (*n* = 5; unpaired *t* test). (**C**) Western blot of DNM2 in muscle from a patient with the *DNM2* S619L mutation compared with control. ns, not significant comparing WT vs. *Dnm2*^SL/+^. Charts show individual points, with additional lines indicating mean ± SEM.

To create a stable mammalian model for the severe ADCNM form, we engineered a transgenic mouse line through homologous recombination in C57BL/6N embryonic stem (ES) cells (Figure 1, A and B). Both heterozygous male and female *Dnm2*^SL/+^ mice were fertile and were crossed with WT and *Dnm2*^SL/+^ mice to generate the disease model (*Dnm2*^SL/+^ mice) and homozygous *Dnm2*^SL/SL^ mice. Genotypes were confirmed by PCR and DNA sequencing (Figure 1, C and D). At postcoitum day 18.5 (18.5 dpc), the expected Mendelian segregation was observed, while no homozygous mice survived to P2, and a partial mortality of the heterozygous was noted between 18.5 dpc and day 10 (Figure 1E). Homozygous mice that were identified all died within the first hours of birth. After day 10, all *Dnm2*^SL/+^ mice survived up to at least 18 months (18 mo). In the surviving *Dnm2*^SL/+^ mice, DNM2 protein level was increased by around 2-fold in tibialis anterior (TA) muscle compared with WT littermates (Figure 2A and Supplemental Figure 5; supplemental material available online with this article; https://doi.org/10.1172/jci.insight.137899DS1). Quantitative PCR (qPCR) analysis of the ubiquitous *Dnm2* isoform (Ub-*Dnm2*) from TA muscles revealed no significant differences between *Dnm2*^SL/+^ and WT mice (Figure 2B). In addition to Ub-*Dnm2*, we previously reported the presence of a skeletal muscle specific isoform (M-*Dnm2*) containing exon12b encoding for 10 amino acids located between the stalk and PH domains. M-*Dnm2* represents about 71% of all *Dnm2* isoforms in TA muscle, and it was slightly decreased in the *Dnm2*^SL/+^ muscle. In a 17-month-old patient muscle biopsy that was accessible, DNM2 antibody detected a protein at the expected size and higher molecular weight signals, suggesting possible aggregation or modification specifically of the S619L protein (Figure 2C). In conclusion, the S619L *Dnm2* mutation is incompatible with postnatal life at the homozygous state and affects the survival of heterozygous mice, the latter representing the disease model for ADCNM.

### Dnm2^SL/+^ mice develop a severe muscle phenotype resembling centronuclear myopathy.

**Figure 3:**
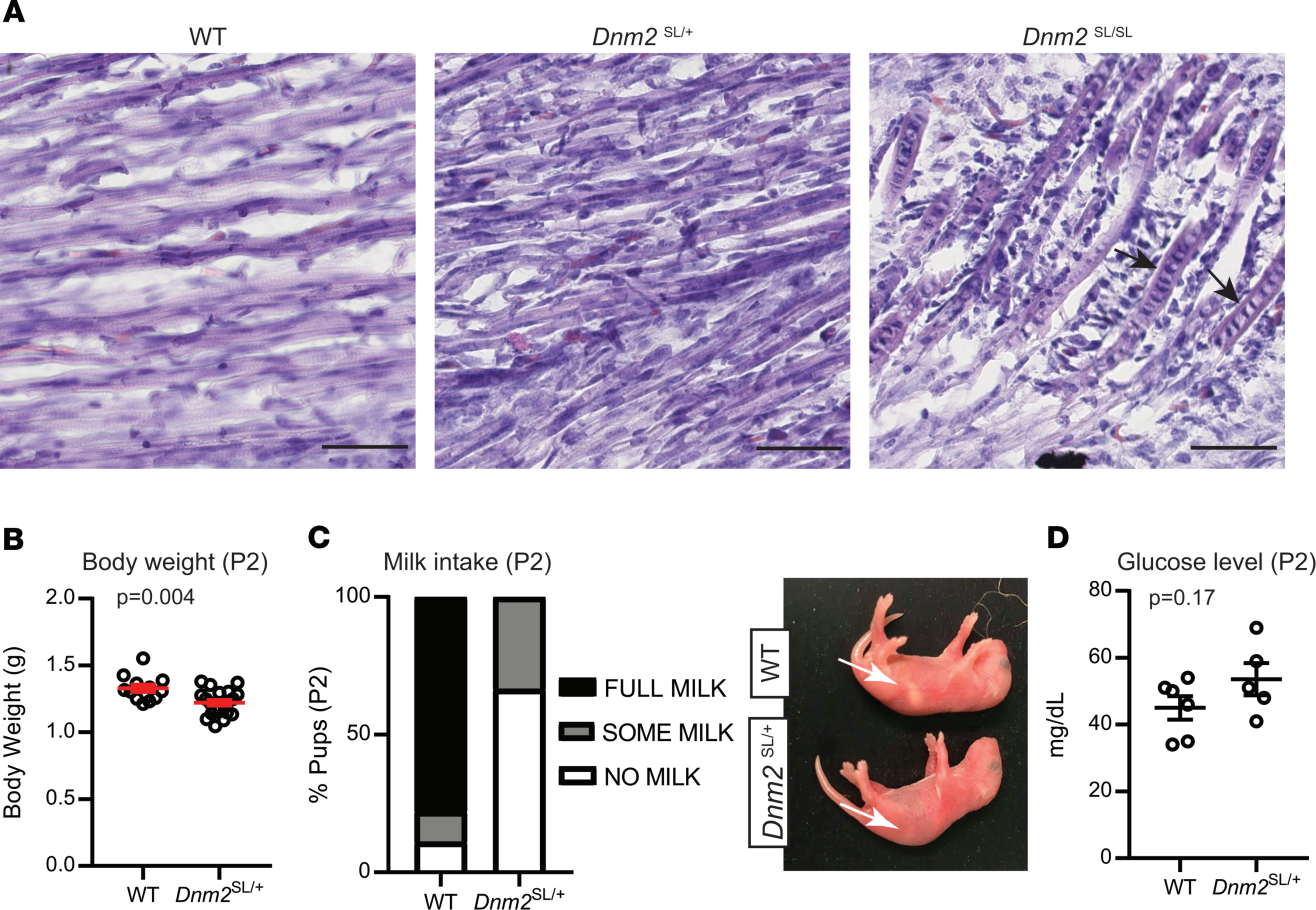
*Dnm2*^SL/+^ pups display reduced body weight and delayed muscle maturation. (**A**) H&E staining of lower limb muscles from 18.5 dpc WT, *Dnm2*^SL/+^, and *Dnm2*^SL/SL^ embryos. Arrows point to myonuclei chains. Scale bar: 50 μm. (**B–D**) Body weight (12 ≤ *n* ≤ 20; unpaired *t* test) (**B**), milk intake (graph shows average for each group and genotype, 12 ≤ *n* ≤ 20) (**C**), and glucose level in blood (5 ≤ *n* ≤ 6; unpaired *t* test) (**D**) in day 2 (P2) WT and *Dnm2*^SL/+^ pups. Charts show individual points, with additional lines indicating mean ± SEM.

**Figure 4:**
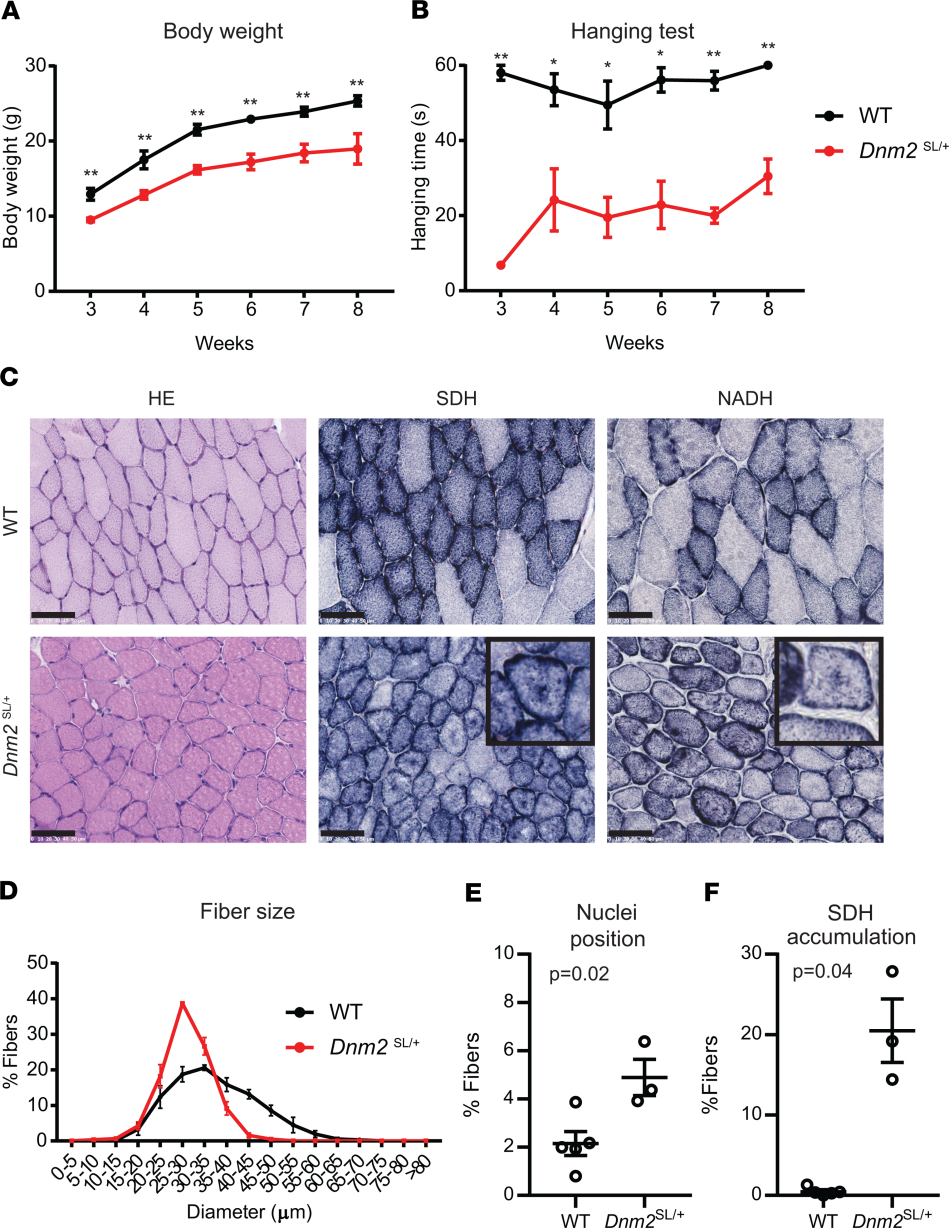
*Dnm2*^SL/+^ develop a severe muscle phenotype resembling centronuclear myopathy. (**A** and **B**) Body weight (*n* = 5; unpaired *t* test for each individual time point) (**A**) and hanging test performance (*n* = 5; unpaired *t* test for each individual time point) (**B**) between 3 and 8 weeks in WT and *Dnm2*^SL/+^ mice. Maximum hanging time is 60 seconds. Both graphs show average value for each time point and genotype, with additional lines indicating mean ± SEM. (**C**) H&E, succinate dehydrogenase (SDH), and reduced nicotinamide adenine dinucleotide (NADH) staining of TA muscle sections from WT and *Dnm2*^SL/+^ mice at 8w (*n* = 5 per staining and group). Note the central accumulation of SDH stain specifically in the *Dnm2*^SL/+^ muscle. Scale bar: 50 μm. (**D–F**) Quantifications of fiber size (3 ≤ *n* ≤ 5, graph shows average value ± SEM for each diameter and genotype) (**D**), nuclei internalization and centralization (3 ≤ *n* ≤ 5; unpaired *t* test) (**E**), and percentage of fibers with central accumulation of SDH stain (3 ≤ *n* ≤ 5; Welch’s *t* test) (**F**). **P < 0.01; *P < 0.05 comparing WT vs. *Dnm2*^SL/+^. Charts show individual points, with additional lines indicating mean ± SEM unless differently stated.

To investigate if the *Dnm2*^SL/+^ mouse line represents a faithful model for severe ADCNM, mice were characterized at different time points, from 18.5 dpc to adulthood, at the clinical and histological levels. At 18.5 dpc, while there was no obvious difference in myofiber size and general organization between WT and *Dnm2*^SL/+^ mice by H&E of lower limb muscles, abnormal nuclei shape, and central chains of nuclei were seen in the homozygous mice (Figure 3A). Central chains of nuclei are often seen in CNM patient myofibers (9). At day 2, body weight was already significantly decreased in *Dnm2*^SL/+^ mice (Figure 3B). We thus explored feeding ability and identified a decrease in milk intake in the stomach, albeit with unchanged blood glucose levels (Figure 3, C and D). These findings suggest a feeding defect causing decreased body weight. Body weight in males was below WT levels over time up to 8w, when mice were sacrificed for further analyses (Figure 4A). A very strong decrease in hanging ability was noted at all time points analyzed in *Dnm2*^SL/+^ mice, reflecting a major impairment in motor function (Figure 4B). ADCNM displays specific histological hallmarks: abnormal oxidative staining, centralized nuclei, and fiber size heterogeneity, which are the key for diagnosis (9). H&E staining of TA muscles at 8w revealed smaller and rounder muscle fibers in *Dnm2*^SL/+^ mice (Figure 4, C–E). There was a slight tendency for increased internal or central nuclei. In addition, succinate dehydrogenase (SDH) and reduced nicotinamide adenine dinucleotide (NADH) staining confirmed fiber hypotrophy and highlighted abnormal accumulation of oxidative activity in the center of fibers (Figure 4, C and F). Overall, the *Dnm2*^SL/+^ mouse displayed a severe motor defect and a CNM-like histology, validating this model as faithfully reproducing the severe form of ADCNM.

### Pathological mechanisms at the basis of ADCNM.

**Figure 5:**
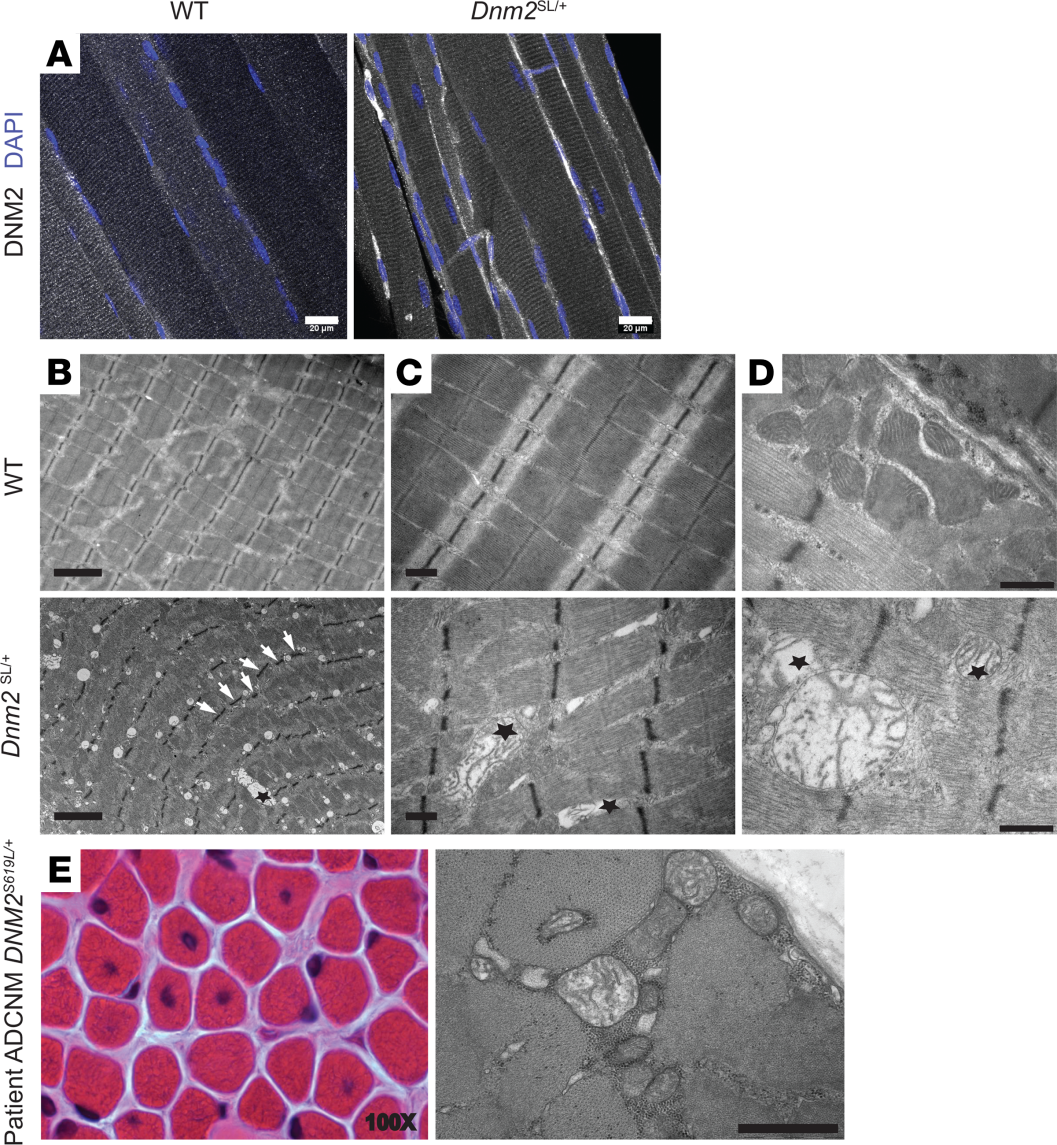
Abnormal mitochondria structure in the *Dnm2*^SL/+^ mice. (**A**) Subcellular localization of DNM2 by immunofluorescence in longitudinal sections from TA muscles at 8w. Scale bar: 20 μm. (**B**) Ultrastructural analysis by electron microscopy in WT and *Dnm2*^SL/+^ TA muscles at 8w (*n* = 2 replicates per group). Arrows point to Z-line misalignment, and stars indicate swollen mitochondria. Scale bar: 2 μm. (**C** and **D**) Higher magnification showing swollen mitochondria with disrupted cristae in the *Dnm2*^SL/+^ muscle only. Scale bars: 0.5 μm (**C**) and 500 nm (**D**). (**E**) Electron microscopy (Scale bar: 2 μm) and H&E (100× magnification) representative images from a muscle from a patient carrying the S619L mutation.

To decipher the defects at the basis of the disease, we first checked the localization of DNM2 in muscle. In WT and *Dnm2*^SL/+^ muscles, DNM2 displayed a striated pattern reminiscent of the sarcomeric organization and fitting with Z-line localization, as previously reported (Figure 5A) (14). The S619L mutation did not obviously affect this localization. Ultrastructural analyses with transmission electron microscopy showed slight misalignment of the Z-line (Figure 5B). The main defect was the presence of vacuole-like structures that were confirmed to be mitochondria on higher magnification. While well localized between the sarcomeres at the A band, mitochondria were often enlarged and rounded and were devoid of most cristae (Figure 5, C and D, and Supplemental Figure 1). Mitochondrial structure was normal in the muscles of the reported *Dnm2*^RW/+^ mouse, supporting that the *Dnm2*^SL/+^ mouse represents a more severe form of myopathy than the *Dnm2*^RW/+^ mouse (17). We did not detect the same type of structural defect in liver and heart tissues, only a decrease in mitochondrial area in liver (Supplemental Figure 2). Based on these findings, we analyzed the muscle of an ADCNM patient with the DNM2 S619L mutation by electron microscopy and found similar enlarged mitochondria devoid of most cristae, highlighting an overlooked phenotype in patients (Figure 5E). Other structural alterations found in other congenital myopathies such as cores or rods were absent.

Zebrafish or mice overexpressing the S619L mutant displayed a disrupted distribution and structural fragmentation of the NMJ (15, 16). However, the transgenic *Dnm2*^SL/+^ mouse has a normal distribution and NMJ area (Supplemental Figure 3), indicating that defects of the NMJ are not major hallmarks of the disease and are probably due to acute overexpression. As different DNM2 mutations are linked to peripheral CMT neuropathy, peripheral nerves were investigated. No structural defect in the sciatic nerve was detected in the *Dnm2*^SL/+^ mouse (Supplemental Figure 4). Taken together, the most prominent feature was a strong impairment in mitochondria in muscle, which is expected to impact muscle performance.

### The defects in muscle force of the Dnm2^SL/+^ mouse are reverted upon DNM2 reduction.

**Figure 6:**
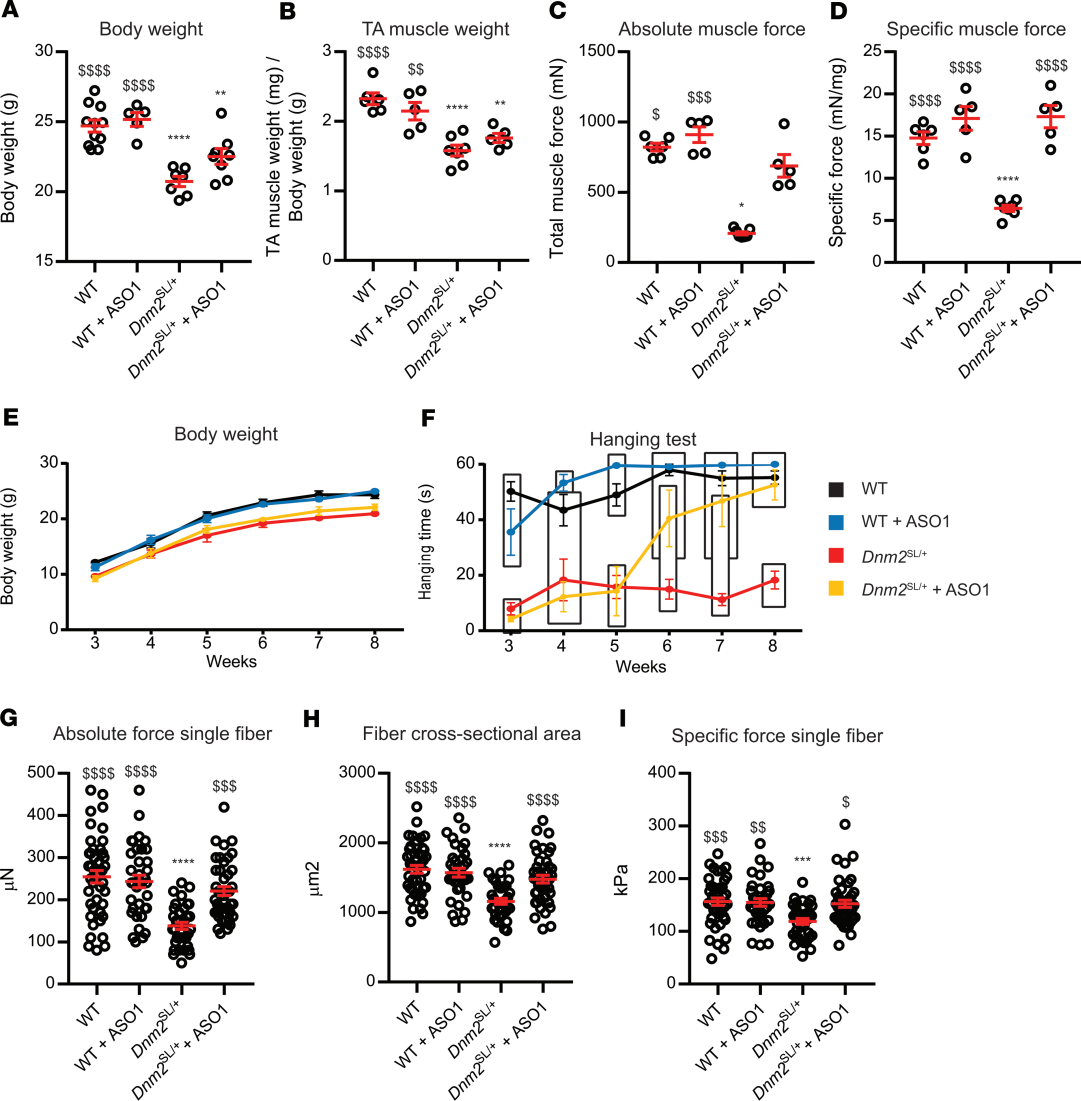
DNM2 reduction reverts the motor defects of the *Dnm2*^SL/+^ mice. (**A–D**) Body weight (5 ≤ *n* ≤ 11; 1-way ANOVA with Tukey’s post hoc test) (**A**), TA muscle weight (5 ≤ *n* ≤ 7; 1-way ANOVA with Tukey’s post hoc test) (**B**), absolute muscle force (5 ≤ n ≤ 7; Kruskal-Wallis test with Dunn’s post hoc test) (**C**), and specific muscle force (5 ≤ *n* ≤ 7; 1-way ANOVA with Tukey’s post hoc test) (**D**) measured at 8w in WT and *Dnm2*^SL/+^ mice treated or not with ASO-1 antisense oligonucleotide targeting *Dnm2*. (**E** and **F**) Body weight and hanging performance of WT and *Dnm2*^SL/+^ mice treated or not with ASO-1 between 3 and 8 weeks (6 ≤ *n* ≤ 7). Blocks show groups with nonsignificant difference in hanging test performance (1-way ANOVA with Tukey’s post hoc test for each individual time point). Note the hanging performance of the treated *Dnm2*^SL/+^ mice was not statistically significant from WT from week 6. Both curves show average values ± SEM for each time point and genotype. (**G–I**) Absolute muscle force (Kruskal-Wallis test with Dunn’s post hoc test) (**G**), fiber cross-sectional area (1-way ANOVA with Tukey’s post hoc test) (**H**), and specific muscle force (Kruskal-Wallis test with Dunn’s post hoc test) (**I**) measured in single myofibers from 8-week-old WT and Dnm2^SL/+^ mice treated or not with ASO-1 antisense oligonucleotide targeting Dnm2 (35 ≤ *n* ≤ 43). ****P < 0.0001 and **P < 0.01 comparing vs. WT. ^$$$$^*P* < 0.0001; ^$$$^*P* < 0.001; ^$$^*P* < 0.01; ^$^*P* < 0.05 vs. *Dnm2*^SL/+^. Charts show individual points, with additional lines indicating mean ± SEM unless differently stated.

To further characterize the impact of the S619L mutation and test a potential therapeutic approach, we analyzed an independent cohort of mice with or without injections of ASO targeting the *Dnm2* pre-mRNA and mature mRNA. Reduction of DNM2 upon injection of ASO-1 was shown to prevent and revert the phenotypes of the X-linked CNM model, the *Mtm1*^y/–^ mouse (32). This strategy was also applied to the *Dnm2*^RW/+^ mouse and appeared to ameliorate the mild muscle phenotypes of this model (22). In this last study, ASO injections were done before disease onset, preventing a full reversion study. Here, weekly i.p. injections of 25 mg/kg ASO-1 were performed in *Dnm2*^SL/+^ mice from 3w to 7w and analyzed at 8w. Of note, before 3w, *Dnm2*^SL/+^ mice displayed feeding defects, reduced body and muscle weight, and strongly impaired motor performance (Figure 3 and Figure 4). Upon treatment, the differences in body and muscle weights compared with treated or untreated WT mice were decreased, albeit not fully normalized (Figure 6, A and B). The untreated *Dnm2*^SL/+^ mice displayed a strong decrease in absolute and specific TA muscle force, while the treated animals had a specific force back to WT level after 5w of treatment (Figure 6, C and D). Untreated *Dnm2*^SL/+^ mice had a strongly impaired motor performance at all ages, while it was normalized after 3w of treatment (Figure 6, E and F, and Supplemental Movie 1). To assess if the force decrease was due to an intrinsic defect of myofibers, isolated myofibers were analyzed. The absolute force, cross-sectional area (CSA), and specific force of myofibers from the TA muscle of *Dnm2*^SL/+^ mice were all significantly decreased, while treatment for 5w normalized these parameters (Figure 6, G–I). Overall, DNM2 reduction with ASO targeting *Dnm2* efficiently reverted both locomotor and force defects of the *Dnm2*^SL/+^ mouse, supporting that the mutation has a gain-of-function activity in vivo.

### CNM histological hallmarks of the Dnm2^SL/+^ mouse are greatly ameliorated upon DNM2 reduction.

**Figure 7:**
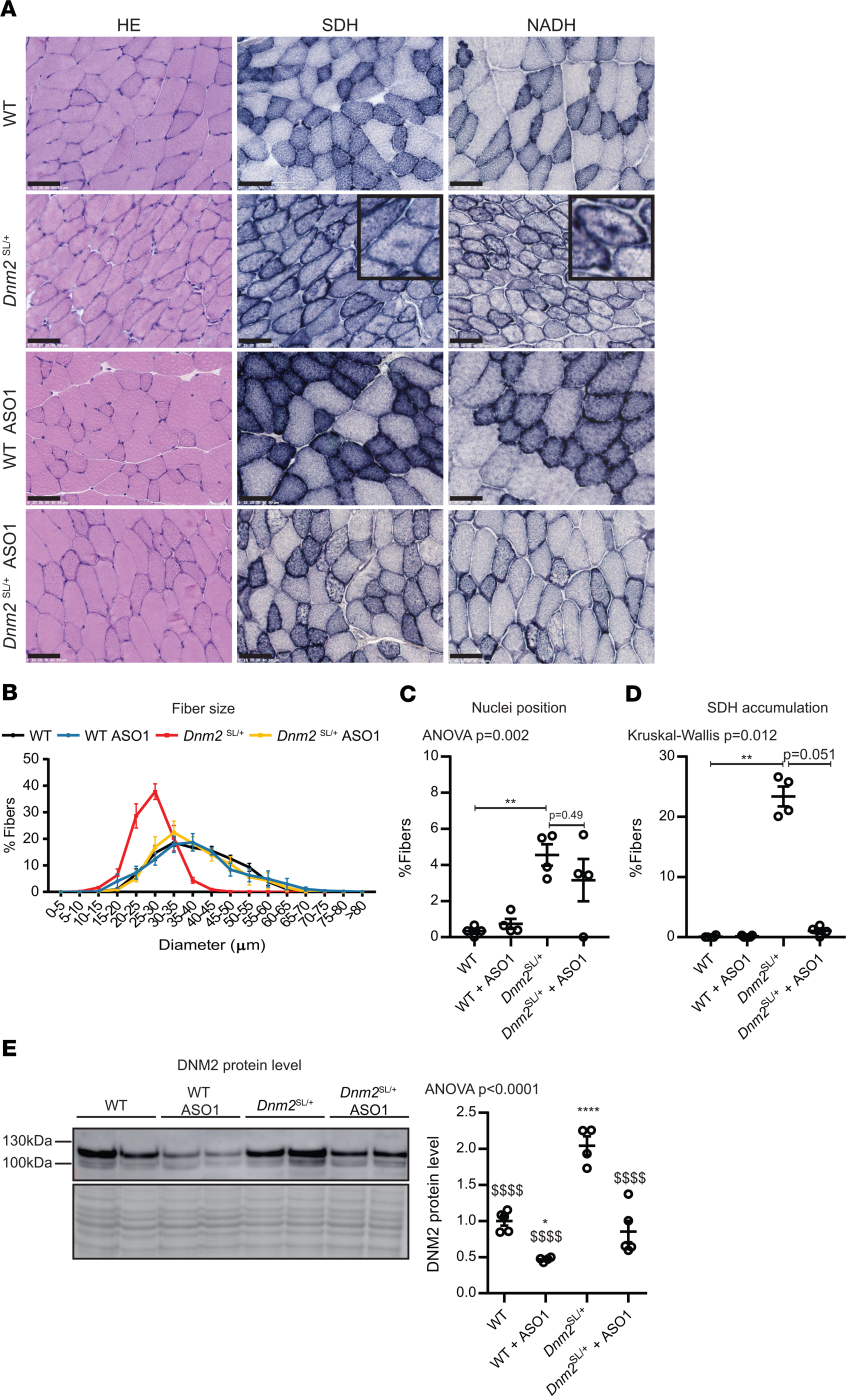
CNM histological hallmarks of *Dnm2*^SL/+^ mice are ameliorated upon DNM2 reduction. (**A**) H&E, SDH, and NADH stainings of TA muscle sections from 8-week-old WT and *Dnm2*^SL/+^ mice treated or not with ASO-1. Scale bar: 50 μm (*n* = 5 per group and staining). Inset, 2× magnification. (**B–D**) Quantification of fiber size (*n* = 4, graph shows average value ± SEM for each diameter and genotype) (**B**), nuclei internalization and centralization (*n* = 4) (**C**), and percentage of fibers with central accumulation of SDH stain (*n* = 4) (**D**). (**E**) DNM2 protein level in TA muscles of 8-week-old WT and *Dnm2*^SL/+^ mice treated or not with ASO-1 (4 ≤ *n* ≤ 5; 1-way ANOVA with Tukey’s post hoc test). ****P < 0.0001 and **P < 0.01 comparing vs. WT. ^$$$$^*P* < 0.0001 vs. *Dnm2*^SL/+^. Charts show individual points with additional lines indicating mean ± SEM unless differently stated.

We next investigated if the rescue of the muscle force correlated with an improvement of the CNM-like histology by staining TA muscles with H&E, SDH, and NADH. The decreased fiber size in untreated *Dnm2*^SL/+^ mice was fully normalized upon ASO-1 injections (Figure 7, A and B). Due to the weak defects in nuclei localization, it was difficult to conclude on an improvement (Figure 7C). The oxidative staining was greatly ameliorated in treated *Dnm2*^SL/+^ mice, with near complete loss of the abnormal central accumulation; however, some fibers presented a heterogeneous staining unlike WT (Figure 7, A and D). Western blotting with DNM2 antibody of TA muscle lysates showed that ASO-1 treatment efficiently reduced DNM2 protein levels (Figure 7E and Supplemental Figure 5). While untreated *Dnm2*^SL/+^ mice displayed increased DNM2 levels, the reduction of DNM2 down to the WT level correlated with the phenotypic rescue. We conclude that DNM2 reduction corrected the CNM-like histological abnormalities of the *Dnm2*^SL/+^ mouse.

**Figure 8:**
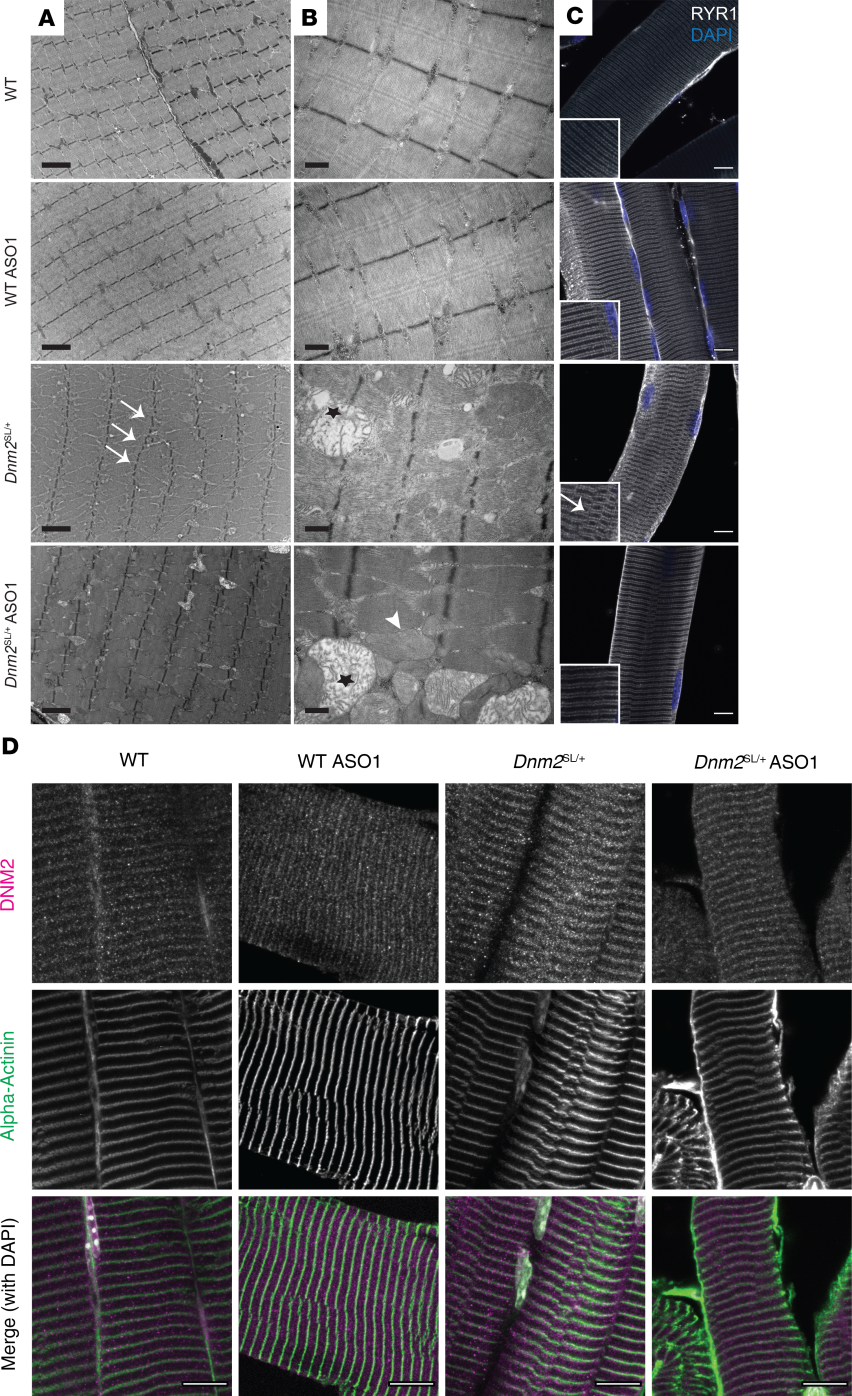
Ultrastructural analysis of *Dnm2*^SL/+^ muscles upon DNM2 reduction. (**A**) Ultrastructural view by electron microscopy in TA muscles from 8-week-old WT and *Dnm2*^SL/+^ treated or not with ASO-1 antisense oligonucleotide targeting *Dnm2* (*n* = 2). Arrows point to Z-line misalignment, and stars indicate swollen mitochondria. Scale bar: 2 μm. (**B**) Higher magnification on mitochondria showing 2 populations in the ASO-1 treated *Dnm2*^SL/+^ mice: swollen mitochondria with disrupted cristae (indicated by a star) and normal mitochondria (indicated by arrowhead). Scale bar: 0.5 μm. (**C** and **D**) Immunofluorescence in TA muscles from 8-week-old mice (*n* = 2) of RYR1 (ryanodine receptor, a marker of sarcoplasmic reticulum at the triad) and DAPI for nuclei (arrow points to misalignment) (**C**) and of DNM2 together with α-actinin (a marker of Z-line) and DAPI for nuclei (**D**). Scale bar: 10 μm.

We uncovered sarcomeric misalignment and a strong mitochondrial structural defect in the *Dnm2*^SL/+^ mice (Figure 5). Electron microscopy revealed an improvement of the sarcomeric alignment upon treatment, and this was confirmed by immunolabeling of α-actinin and the RYR1 calcium channel, markers of Z-line and sarcoplasmic reticulum, respectively (Figure 8, A, C, and D). We observed 2 populations of mitochondria: enlarged mitochondria with highly perturbed cristae structure, as in the untreated, and normal or slightly enlarged mitochondria with normal cristae close to WT conditions (Figure 8B). The second population was not seen in untreated animals, suggesting an improvement in mitochondrial structure upon DNM2 reduction. Overall, ASO treatment had a positive impact on the main structural defects observed on mitochondria and sarcomere, correlating with the locomotor and force correction.

## Discussion

Here, we created and validated the first mammalian model to our knowledge for the severe form of ADCNM due to DNM2 mutations. In particular, we focused on the S619L missense mutation, affecting a residue strongly implicated in the regulation of the PH domain conformational switch that regulates the self-assembly, membrane binding, and fission of dynamin. *Dnm2*^SL/+^ mice display an early and severe motor defect linked to force reduction and mitochondria structural anomalies that faithfully reproduce the phenotype of CNM patients, including the histological hallmarks of their muscles. Reduction of DNM2 level with ASO promoting the degradation of *Dnm2* pre-mRNA after disease onset efficiently rescued the main behavior and histological phenotypes and reverted the early signs of the disease.

### A faithful mammalian model for severe centronuclear myopathy linked to DNM2 mutation.

The *Dnm2*^SL/+^ mice, like the ADCNM severe form, have muscle weakness from birth (10, 11). Patients were reported to have hypotonia with weak suckling, and the *Dnm2*^SL/+^ mice had neonatal feeding defects. *Dnm2*^SL/+^ muscle showed most of the hallmarks of patient histology, including fiber hypotrophy and central accumulation of oxidative staining. However, centralization of nuclei was barely observed in adult mice; the *Dnm2*^RW/+^ mouse did not show any nuclei internalization, suggesting that this phenotype in ADCNM is difficult to reproduce in mice (17). Conversely, in the *Dnm2*^SL/+^ mouse at 18.5 dpc, central chains of nuclei were often seen as in CNM patient myofibers (9). In addition, we uncovered swollen mitochondria with altered cristae in the *Dnm2*^SL/+^ mouse, leading to a reinvestigation of human muscle in which we confirmed this phenotype. The different phenotypes in the *Dnm2*^SL/+^ mice are much more pronounced and of earlier onset than in the *Dnm2*^RW/+^ mice, confirming the genotype-phenotype correlation observed in patients (10). Moreover, it makes the *Dnm2*^SL/+^ mice a better and more attractive model for testing therapies.

The S619L mutated DNM2 was previously overexpressed in *drosophila*, zebrafish, and mice. Noteworthy, overexpression of DNM2 by itself could trigger cellular defects, as noted when WT-DNM2 was overexpressed in WT mice (14, 27). In both *drosophila* and zebrafish, only the larva stage could be explored, as *drosophila* expressing the S619L mutant could not hatch and only transient overexpression was achieved in zebrafish through RNA injection (12, 13, 18). In mice with exogenous overexpression of the S619L mutant, the development of a CNM disease could not be followed as adult WT mice were injected (16). The *Dnm2*^SL/+^ mouse provides a faithful model to characterize the disease progression and postnatal muscle maturation and function. NMJ defects were reported in zebrafish and mice overexpressing S619L mutant, while we did not observed obvious defect in the *Dnm2*^SL/+^ adult mouse. This difference could be due to the expression level of the S619L mutant or to a compensatory mechanism in mice. In accordance to the last hypothesis, some *Dnm2*^SL/+^ mice died by day 10 while all remaining mice survived to at least 18 months. In addition, different DNM2 mutations in the PH domain cause CMT, and it is unclear to date if CNM and CMT phenotypes overlap in the same patient (33, 34). There were no obvious structural defects in the sciatic nerve of the *Dnm2*^SL/+^ mouse, unlike the axonal degeneration and loss of myelinating fibers seen in CMT patients (35), suggesting no overlap.

### Physiological impact of dynamin dysregulation.

Previous studies in the *drosophila* and zebrafish overexpressing models reported T-tubule defects that were not evident in the *Dnm2*^RW/+^ mouse and neither in the potentially novel *Dnm2*^SL/+^ mouse (17). In accordance to the mice data, no obvious T-tubule or triad structural defects were reported in patients. However, excitation-contraction coupling was impaired in isolated fibers from the *Dnm2*^RW/+^ mouse, supporting that calcium mishandling could be an important feature in the pathomechanism (36, 37). Here, we uncover a potential cause of the disease, as the main ultrastructural defect was enlarged mitochondria lacking cristae. Swollen mitochondria harboring disorganized cristae were also reported in a skeletal muscle–specific KO, suggesting that the mitochondria defects observed in the *Dnm2*^SL/+^ mouse originate from the muscle (38). DNM2 was recently suggested to fission mitochondria in cooperation with another dynamin, DRP1 (39); however, this point is highly controversial (40, 41), and we cannot exclude that the impact of DNM2 alteration on mitochondria in muscle is indirect. The movement of the PH domain was shown to be crucial for regulating self-assembly, membrane binding, and fission, and the S619L mutation destabilizes this conformational switch in vitro (24). The physiological relevance of this process was unknown, and preliminary data in cultured cells reveal that the S619L mutation only slightly delayed clathrin-mediated endocytosis (31). In DNM2-depleted HeLa cells, DNM2 S619L reexpression has been reported to slightly increase clathrin-mediated endocytosis of transferrin (28). Our present data support that this conformational switch is of main importance, specifically for skeletal muscle, despite the ubiquitous expression of DNM2. Of note, skeletal muscles specifically contain the M-DNM2 isoform, with exon12b encoding for 10 amino acids in close proximity to the stalk-PH domains interface (42). We thus hypothesize the M-DNM2 is more sensitive to an alteration in the conformational switch of dynamin and that skeletal muscle is more sensitive to CNM mutations, underlying the fact that CNM patients appear specifically affected in skeletal muscle despite the ubiquitous expression of *DNM2*. Taken together, while the main function of classical dynamins described in cultured cells is vesicle fission in clathrin-mediated endocytosis, the role of DNM2 in muscle may primarily involve other mechanisms in addition to endosome fissioning.

### Proof of concept for phenotypic reversion of the severe ADCNM.

As the *Dnm2*^SL/+^ mouse displays a faithful and severe phenotype, it is an attractive model for testing therapies, especially those directed toward the severe CNM form. Here, we tested reducing DNM2 through ASO injection after the onset of phenotypes as a potential therapy for severe ADCNM, as it was reported to be efficient for other CNM forms (32). We found striking improvement of all clinical, histological, and ultrastructural phenotypes upon DNM2 reduction. Strikingly, the disease correction upon DNM2 reduction supports that the phenotypes seen at birth and in early life in affected animals and the structural alterations that may result from a muscle maturation delay or blockade are reversible. Importantly, the motor and force defects reverted back to normal, which was achieved after only 3w of treatment (Figure 6F).These data support further preclinical developments for future clinical trials in patients with *DNM2* mutations and the severe form of ADCNM. Moreover, as ASO leading to DNM2 decrease was able to rescue myotubular myopathy (32), our findings support that a similar treatment can be applied to several myopathies. In addition, the fact that the rescue was obtained in the *Dnm2*^SL/+^ mouse by decreasing overall DNM2 level suggests that impairment of the conformational switch leads to dominant effects. It remains to be determined if specific modulation of this conformational switch will have an impact on the disease.

## Methods

### Animals.

The *Dnm2*^SL/+^ mutant mouse line was created at the Institut Clinique de la Souris (http://www.ics-mci.fr/en/). Briefly, C57BL/6N mouse ES cells were electroporated with a targeting vector carrying the 2 transversions T > C and C > T at positions 1855 and 1856, respectively (NM_001039520.2), and a floxed neomycin resistance cassette with an autoexcission transgene. After G148 selection, different clones were analyzed by PCR and Southern blot using an internal neomycin probe and an external 5′ probe. Before injection into BALB/c blastocysts, the selected clone was karyotyped. The obtained male chimeras were bred with C57BL/6N females. Germline transmission with direct excision of the selection cassette was achieved in the first litter. The following primers were used for genotyping: E forward (Ef): 5′ - CAGAAAGCAGGATCCTCGGTGCC - 3′; E reverse (Er): 5′ - AGTCCAGCTCTGGCTTTGGATCGC - 3′; for sequencing: Mf: 5′ - CCAGAGCCCATGGTCTTAGTGGCC - 3′; Mr: 5′ - ACCCCAGCGCGCAGGAACAG - 3′.

### ASO treatment.

The ASO-1 used in this study to target *Dnm2* was produced by IONIS Pharmaceuticals and validated previously (32). WT and *Dnm2*^SL/+^ mice were treated from 3w to 8w of age with weekly i.p. injections of 25 mg/kg of ASO-1 diluted in sterile PBS. All WT and *Dnm2*^SL/+^ nontreated mice shown in the same graphs with ASO-1–treated mice were injected with an equivalent volume of sterile PBS.

### Phenotyping.

From 3w to 8w, mice were phenotyped once per week. Body weight and whole body hanging ability were measured. Hanging test was performed by suspending the mice in a cage lid then turned upside down for up to 60 seconds, and the time to fall was recorded. This experiment was repeated 3 times by each mouse, with a 5-minute interval to allow a recovery period.

### Muscle contractile properties.

TA muscle contraction properties were evaluated by measuring in situ muscle contraction after sciatic nerve stimulation using the Complete1300A mouse Test System (Aurora Scientific) as described previously (43). Mice were anesthetized by sequential i.p. injections of domitor/fentanyl mix (2/0.28 mg/Kg), diazepam (8 mg/Kg), and fentanyl (0.28 mg/Kg). Afterward, TA distal tendon was detached and tied to an isometric transducer. The sciatic nerve was then stimulated by pulses of 1–125 Hz, and the absolute maximal force was determined. The specific maximal force was obtained by dividing the absolute muscle force by the TA muscle weight.

### Single myofiber force production.

On the day of the experiment, single myofibers were dissected from TA bundles in a relaxing solution. They were then individually attached between connectors leading to a force transducer and a lever arm system (model 1400A; Aurora Scientific). Sarcomere length was set to about 2.50 μm, and the temperature was set to 15°C. Fiber CSA was estimated from the width and depth, assuming an elliptical circumference. The absolute maximal isometric force generation was calculated as the difference between the total tension in the activating solution (pCa 4.50) and the resting tension measured in the same myofiber while in the relaxing solution (pCa 9.0). Specific force was defined as absolute force divided by CSA. Relaxing and activating solutions contained 4 mM Mg-ATP, 1 mM free Mg^2+^, 20 mM imidazole, 7 mM EGTA, 14.5 mM creatine phosphate, and KCl to adjust the ionic strength to 180 mM and pH to 7.0. The concentrations of free Ca^2+^ were 1 × 10^–9^ M (relaxing solution) and 1 × 10^–4.5^ M (activating solution).

### Muscle histology.

TA muscles were dissected and weighted. Muscles were then frozen in liquid nitrogen–cooled isopentane and stored at –80°C for H&E, SD,H and NADH histology analysis. Transversal cryosections (8 μm) were prepared and stained. They were observed using the Hamamatsu 322 NanoZoomer 2HT slide-scanner.

### Protein extraction and Western blot.

TA muscles were lysed in RIPA buffer supplemented with PMSF 1 mM and complete mini-EDTA free protease inhibitor cocktail (Roche Diagnostics) using a Precellys 24 tissue homogenizer (Bertin Technologies). Protein concentrations were calculated using the Bio-Rad Protein Assay Kit. Protein (20μg) in 5× Lane Marker Reducing Buffer (Thermo Fisher Scientific) were separated in 10% SDS-PAGE gel. The gel was transferred on nitrocellulose membrane using Transblot Turbo RTA transfer kit (Bio-Rad) for 7 minutes at 2.5A. Protein loading was determined by Ponceau S staining. Membranes were blocked for 1 hour in TBS containing 5% nonfat dry milk and 0.1% Tween-20 before a 2-hour room-temperature or overnight 4°C incubation with DNM2 primary antibody (2865, 1/700 described in ref. 14). Subsequently, membranes were incubated with secondary antibody coupled to horseradish peroxidase. Nitrocellulose membranes were visualized in Amersham Imager 600 (GE Healthcare Life Sciences).

### RNA extraction and qPCR.

RNA was isolated from TA muscle using TRI Reagent (Molecular Research Center). cDNA synthesis was achieved using SuperScriptIV Reverse Transcriptase (Thermo Fisher Scientific). qPCR was done with cDNA amplified and SYBER Green Master Mix I (Roche Diagnostics) together with 0.1 μM of forward and reverse interexonic primers. Amplicons were analyzed using a Lightcycler 480 (Roche Diagnostics). The following primers were used Ub-*Dnm2* forward: 5′ - ACCTACATCAGGGAGCGAGA - 3′; Ub-*Dnm2* reverse: 5′ - GCTCCTCTGCTGGGCATT - 3′; pan-*Dnm2* forward: 5′ - ACCCCACACTTGCAGAAAAC - 3′; pan-*Dnm2* reverse: 5′ - CGCTTCTCAAAGTCCACTCC - 3′; 12b-*Dnm2* forward: 5′ - ACCTACATCAGGGAGCGAGA - 3′; 12b-*Dnm2* reverse: 5′ - TGTGACCAGCTCCTCAGTATAGA - 3′; *Rpl27* forward: 5′ - AAGCCGTCATCGTGAAGAACA - 3′; *Rpl27* reverse: 5′ - CTTGATCTTGGATCGCTTGGC - 3′ (*Rpl27* primers were described in ref. 44).

### Electron microscopy.

Transmission electron microscopy was performed on TA muscles fixed in 2.5% paraformaldehyde (PFA; Electron Microscopy Sciences), 2.5% glutaraldehyde (Electron Microscopy Sciences), and 50 mM CaCl2 (Sigma-Aldrich) in cacodylate buffer (0.1M, pH = 7.4; Sigma-Aldrich). Muscles were then postfixed in 1% osmium tetroxide in 0.1M cacodylate buffer for 1 hour at 4°C and incubated with 5% uranyl acetate for 2 hours at 4°C. The samples were embedded in Epon 812. Ultrathin sections were cut at 70 nm and contrasted with uranyl acetate and lead citrate; they were finally observed with a Philips CM12 electron microscope equipped with a Gatan OneView Camera (Gatan).

### Immunostaining of muscle longitudinal sections.

For longitudinal immunostaining, TA muscles were fixed as described before (32). After PFA 4% and sucrose 30% incubation, fiber bundles were manually isolated and mounted in SuperFrost Plus adhesion microscopy slides (Thermo Fisher Scientific). Subsequent immunostaining was performed using the following primary antibodies: anti-DNM2 (dilution used 1/100, 2680, described in ref. 14), anti-RYR1 (dilution used 1/100, R129, MilliporeSigma), and anti–α-actinin (dilution used 1/100, A7811, MilliporeSigma). The secondary antibody used was donkey anti–rabbit Alexa 594 (A-21207) or goat anti–mouse Alexa 647 (A-21235) (both Invitrogen), the dilution used was 1/250. To detect NMJ, we used α-Bungarotoxin CF 488A Conjugate (Biotium); the dilution used was 1/1000.

### Statistics.

All data were verified for normal distribution using Shapiro-Wilk test and for homoscedasticity with the Brown-Forsythe test. Normal and homoscedastic data were analyzed using Mann-Whitney *U* test and 2-tailed *t* test, in case of 2-group comparisons, or 1-way ANOVA followed by Tukey’s post hoc test in case of multiple group analyses. For nonnormally distributed or heteroscedastic data, *t* test with Welch’s correction or nonparametric 1-way ANOVA (Kruskal-Wallis) with Dunn’s post hoc test were done, respectively. A *P* value less than 0.05 was considered significant. All statistical tests used were 2-sided. Multiple time points were analyzed separately. Charts show individual points with additional lines indicating mean ± SEM. Curves and graphs were made using GraphPad Prism software.

### Study approval.

Animal care and experimentation were in accordance with French and European legislation and approved by the institutional ethics committee (project nos. 01594.02, 2016052510176016, and 2016031110589922). Mice were kept on 12-hour daylight and 12-hour dark cycle with free access to standard food and water. Lifespan and body weight were followed during this study.

## Author contributions

JL conceived the project. XMM, RSR, JO, NBR, BSC, and JL designed the experiments and analyzed the data. XMM, CK, RSR, JO, and AM performed experiments. XMM and JL wrote the manuscript.

## Supplementary Material

Supplemental data

Supplemental Video 1

## Version 1 08/18/2020

In-Press Preview

## Version 2 09/17/2020

Electronic publication
