# Supplementary material for: Physiological impact and disease reversion for the severe form of centronuclear myopathy linked to dynamin
Source: JCI Insight. 2020 Sep 17;5(18):e137899. doi: 10.1172/jci.insight.137899 (PMC7526554; doi:10.1172/jci.insight.137899)
Supplement: Supplemental data [file jciinsight-5-137899-s020.pdf]

Physiological impact and disease reversion for the severe form of centronuclear myopathy linked to Dynamin

Xènia Massana Muñoz[1,2,3,4], Christine Kretz[1,2,3,4,#], Roberto Silva-Rojas[1,2,3,4,#], Julien Ochala[5], Alexia Menuet[1,2,3,4], Norma B Romero[6,7], Belinda S. Cowling[1,2,3,4,8], Jocelyn Laporte[1,2,3,4,*]

[1] Institut de Génétique et de Biologie Moléculaire et Cellulaire, Illkirch, France

[2] Centre National de la Recherche Scientifique, UMR7104, Illkirch, France

[3] Institut National de la Santé et de la Recherche Médicale, U1258, Illkirch, France

[4] Université de Strasbourg, Illkirch, France

[5] Centre of Human and Applied Physiological Sciences, School of Basic and Medical Biosciences, Faculty of Life Sciences and Medicine, King's College London, SE1 1UL London, United Kingdom

[6] Neuromuscular Morphology Unit, Myology Institute, GHU Pitié-Salpêtrière, Paris, France

[7] Sorbonne Université, AP-HP, INSERM, Centre de référence des maladies neuromusculaires Nord/Est/Ile de France, Paris, France

[8] Dynacure, Illkirch, France

[#] equal contributors

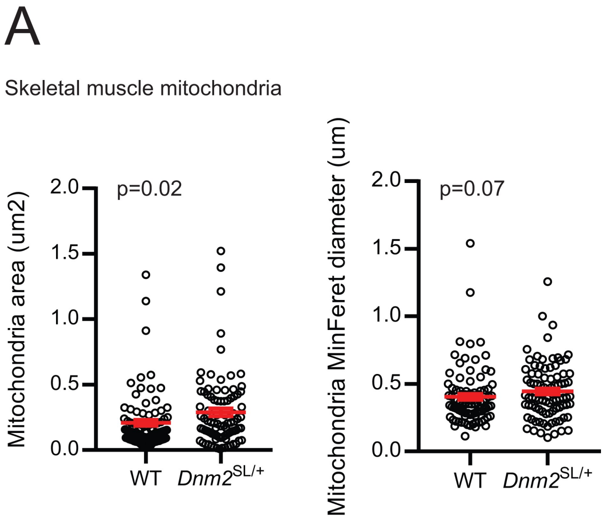

Supplementary Figure 1. Increased area of *Dnm2*$^{SL/+}$ tibialis anterior mitochondria.

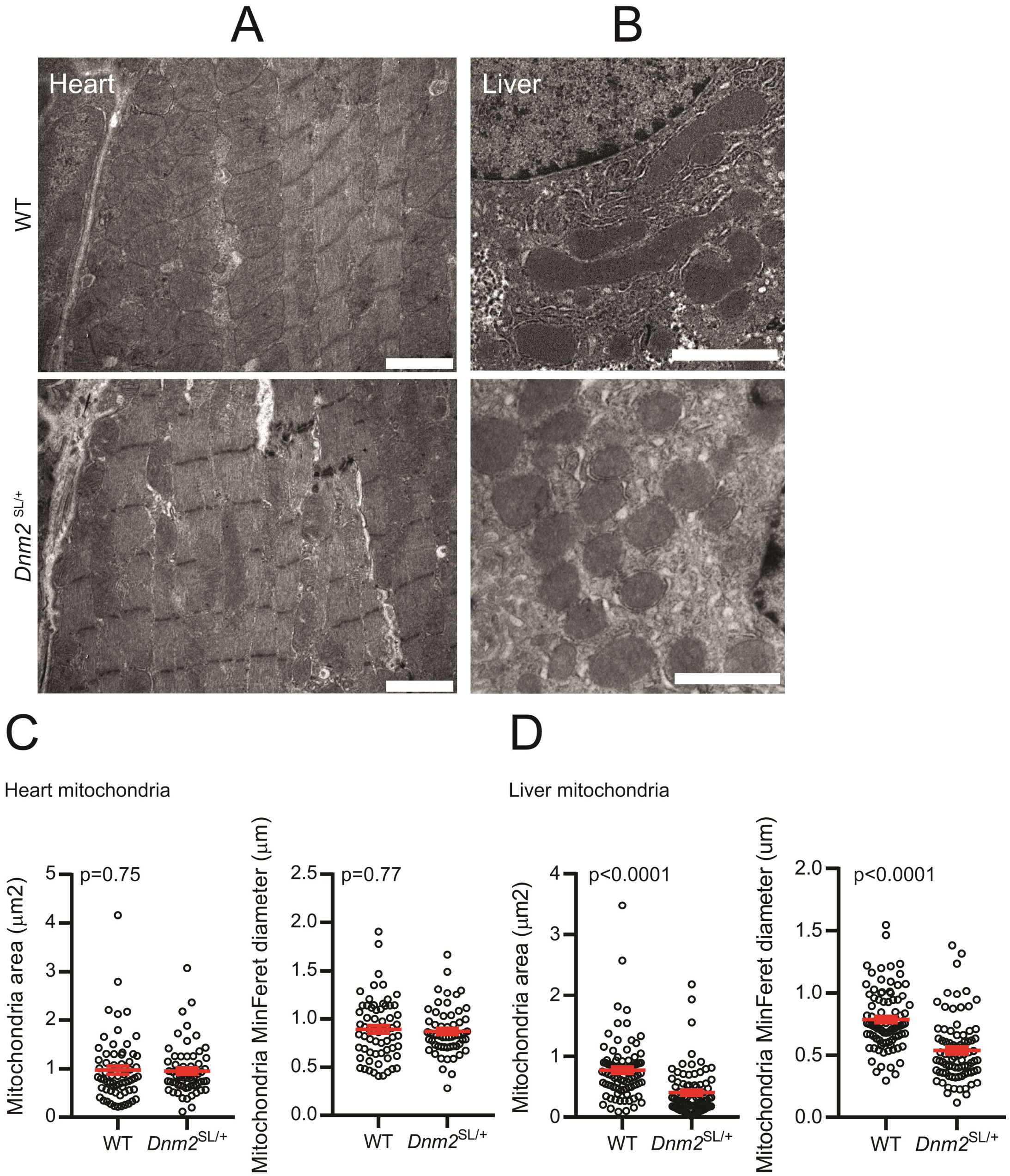

Supplementary Figure 2. Analysis of heart and liver mitochondria. (A-B) Representative images from heart (A) and liver (B) mitochondria (Scalebar = 2µm). (C-D) Quantification of area and Minimum Feret diameter in heart (C) and liver (D) reporting differences in liver mitochondrial size. (In both cases 60≤n≤88 mitochondria (2 animals, 3 different regions of interest), Mann Whitney test).

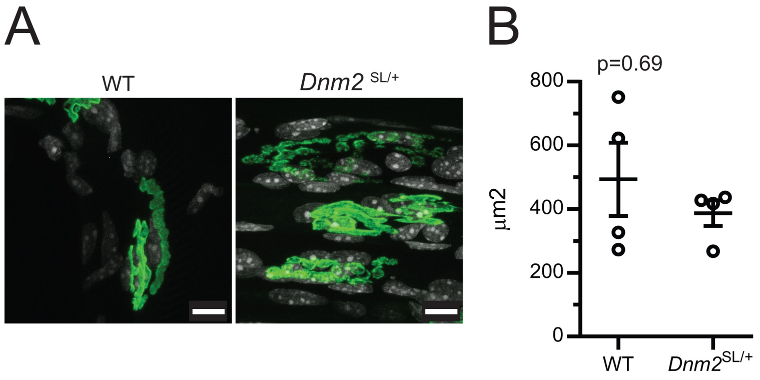

Supplementary Figure 3. Neuromuscular junction staining. (A) Representative images of α-bungarotoxin staining (in green) and DAPI (in gray) in fibers from 8w old WT and *Dnm2*$^{SL/+}$ mice. Scale bar=10μm. (B) Quantification of neuromuscular junction area (n=4).

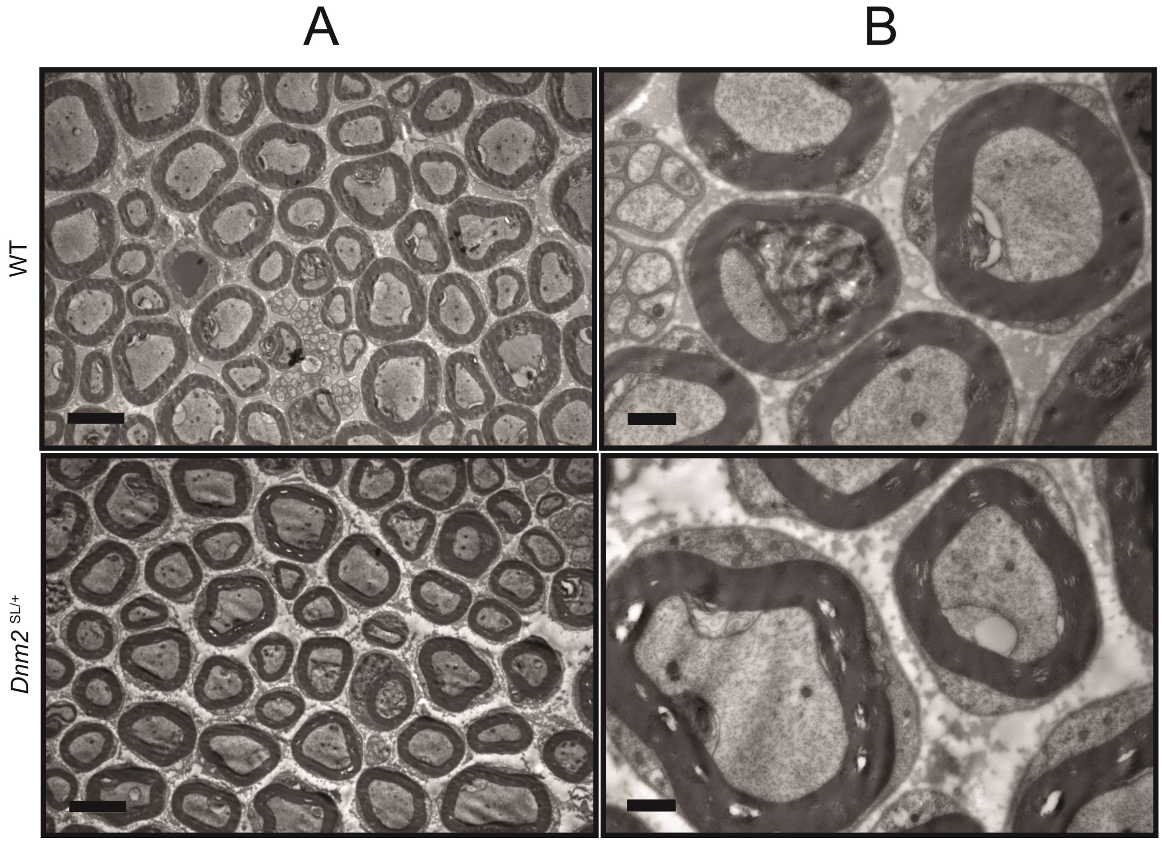

Supplementary Figure 4. Transmission electron microscopy images from sciatic nerve. (A) Transversal sections of sciatic nerve from 8w old WT and *Dnm2*$^{SL/+}$ mice. Scale bar=5μm. (B) Higher magnification of the same sciatic nerves from 8w old WT and *Dnm2*$^{SL/+}$ mice. Scale bar=1μm.

Figure 1F

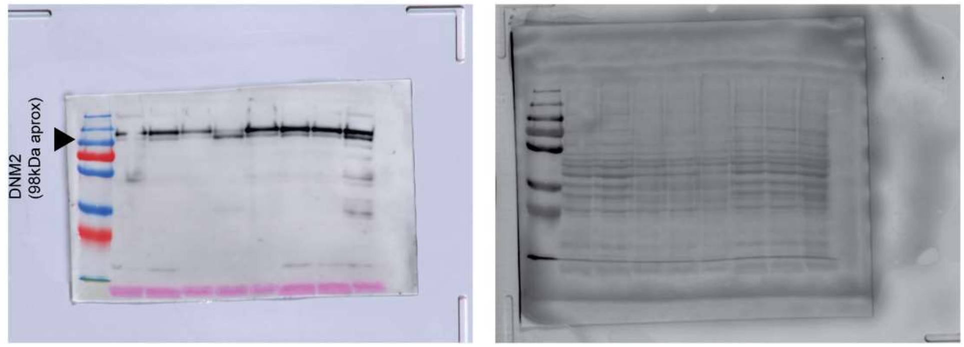

Figure 5H

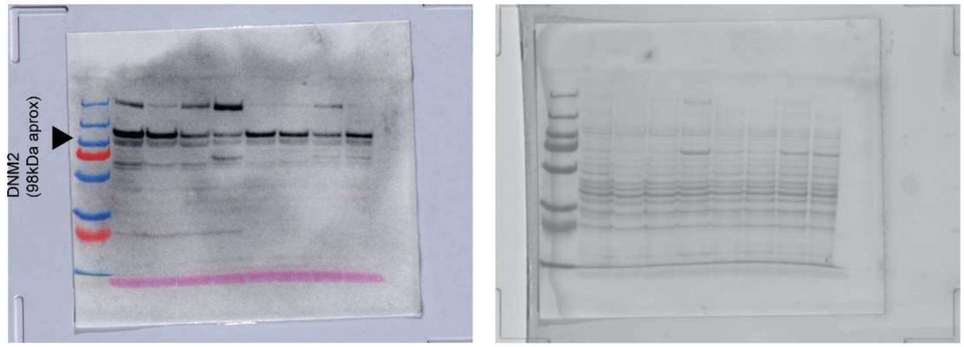

Supplementary figure 5. Uncropped full-length Western blot images.

Supplementary movie 1. Hanging test performance of *Dnm2*$^{SL/+}$ treated with ASO. Video showing two 8 weeks old *Dnm2*$^{SL/+}$ mice untreated and treated with ASO performing hanging test.
